# Are humanized IgE reporter systems potential game changers in serological diagnosis of human parasitic infection?

**DOI:** 10.1007/s00436-021-07352-z

**Published:** 2021-11-12

**Authors:** Prema S. Prakash, Michael H. W. Weber, Jaap J. van Hellemond, Franco H. Falcone

**Affiliations:** 1 Biomedical Research Centre Seltersberg (BFS), Institute for Parasitology, Justus Liebig University Giessen, Giessen, Germany; 2 Department of Medical Microbiology and Infectious Diseases, Erasmus MC University Medical Center, Rotterdam, The Netherlands

**Keywords:** IgE, Diagnosis, RBL reporter system, Luciferase, Fluorescence

## Abstract

Immunoglobulin E (IgE) is thought to have evolved to protect mammalian hosts against parasitic infections or toxins and plays a central role in the pathogenesis, diagnosis, and therapy of IgE-mediated allergy. Despite the prominence of IgE responses in most parasitic infections, and in stark contrast to its use in the diagnosis of allergy, this isotype is almost completely unexploited for parasite diagnosis. Here, we discuss the perceived or real limitations of IgE-based diagnosis in parasitology and suggest that the recent creation of a new generation of very sensitive cellular IgE-based reporters may represent a powerful new diagnostic platform, but needs to be based on a very careful choice of diagnostic allergens.

## The relationship between parasites and IgE is old

IgE is an immunoglobulin isotype only found in mammals (Hellman et al. 2017), where it is thought to have arisen from an early IgY gene duplication event (Warr et al. 1995). IgE is commonly believed to be associated with protection against a range of common parasites (‘worm hypothesis’), such as intestinal worms or blood flukes (Pritchard et al. 2020). An alternative or complementary hypothesis sees the function of IgE as protection against toxins (‘toxin hypothesis’) (Palm et al. 2012). As recently described, the environment at the time the earliest mammalian ancestors evolved some 200 Mio years ago (Hellman et al. 2017) was rich in toxins, parasites, and other allergenic sources (e.g., environmental or food) (Pritchard et al. 2020). This makes it likely that the presence of IgE conferred an evolutionary advantage to the early mammalian ancestors, leading to the preservation of the IgE immune response across all mammals (Pritchard et al. 2020). Therefore, an elevation of parasite-specific and total IgE is a frequent symptom of parasitic infection (Jarrett and Miller 1982), together with peripheral blood eosinophilia (Huang and Appleton 2016) and intestinal mastocytosis (Befus and Bienenstock 1979). The IgE elevation occurs in endoparasitic as well as ectoparasitic infections (or infestations), such as scabies (Arlian et al. 2004). Parasite-specific IgE elevation, however, is not only found in most (if not all) metazoan infections but also in several protozoan infections such as malaria (Perlmann et al. 1994), toxoplasmosis (Sin Yew Wong et al. 1993; Matowicka-Karna and Kemona 2014), leishmaniasis (Atta et al. 1998), or invasive entamoebiasis (Aceti et al. 1989).

In the context of this article, the term ‘allergen’ designates molecules (usually proteins) which are the target of a specific IgE response, i.e., which possess one or more epitopes recognized by IgE. ‘Allergenicity’ here is understood as the ability of multivalent allergens to induce activation of cells bearing the high-affinity IgE receptor FcεRI (mainly mast cells and basophils, but also other leukocyte populations). The intrinsic ability of an allergen to induce a Th2-biased immune response in a naïve host, leading to the synthesis of specific IgE, will not be addressed here. The important property of allergenicity here is the ability of an allergen to engage more than one FcεRI-bound IgE molecule, thereby clustering the receptor and inducing signal transduction, leading to cellular activation and mediator release. In this sense, only molecules that have more than one accessible epitope, within a certain distance of each other, are considered allergenic.

In a primary immune response, IgE is thought to result from either direct IgM➔IgE or indirect, sequential IgM➔IgG➔IgE isotype switching (Xiong et al. 2012). Thus, IgE antibodies take longer to appear in blood than the other isotypes in primary infection. In human experimental infection with the hookworm *Necator americanus*, we were able to detect sensitization of peripheral blood basophils as soon as 5–6 weeks after primary infection by performing basophil activation tests (Falcone et al. 2009). This time point is a bit earlier than the appearance of parasite eggs in the feces of infected individuals known from the work of Geiger and colleagues, where eggs were first detected 68–72 days after infection (Geiger et al. 2008), but closer to the 47 days p.i. described by Ogilvie et al. (1978). Interestingly, and perhaps important in the context of this article, basophil sensitization occurred in the absence of measurable, specific IgE levels in serum (Falcone et al. 2009). A similar situation is seen in the aforementioned work by Ogilvie, where specific IgE levels could only be detected after a third and fourth infection (Ogilvie et al. 1978). The original self-infection report by Ball and Bartlett (1969), however, points to an even earlier possible time point for the appearance of IgE in primary helminth infection. In these experiments, four human volunteers, injected intradermally with serum obtained from the hookworm-infected donor (4 weeks p.i.) and challenged 72 h later with *N. americanus* extracts, all showed very strong Prausnitz–Küstner reactions (a now-disused test in which IgE-containing serum of an allergic donor was injected intradermally into a non-allergic individual). With the caveat of the low number of experimental replicates, this would suggest that anti-parasite IgE responses can become apparent as early as 4 weeks after the original infection, which also matches the earliest time point of basophil conversion found in our work with *N. americanus* (Falcone et al. 2009).

**Fig. 1:**
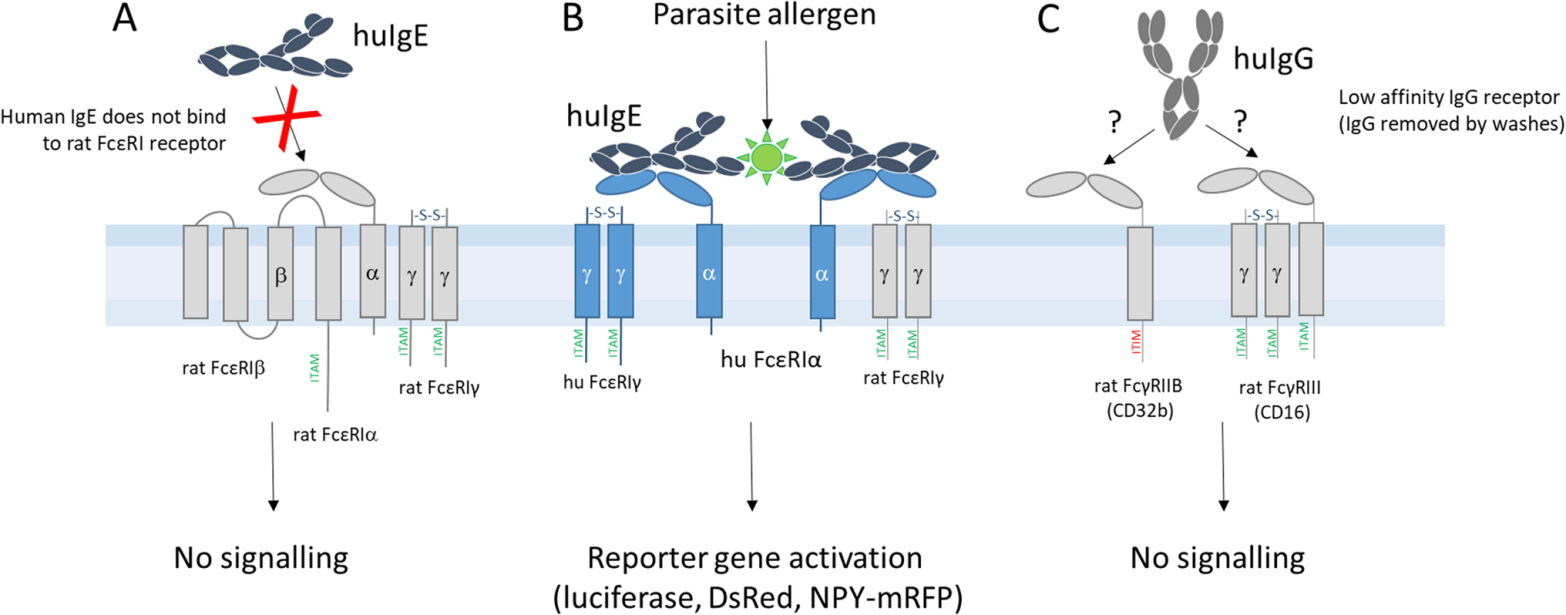
How IgE reporter systems work. Existing IgE reporter systems are based on rat basophilic leukemia (RBL) cells, which are well studied and easy to grow (Falcone et al. 2018). However, because the rat high-affinity IgE receptor does not bind human IgE (**A**) (Miller et al. 1989), they need to be stably transfected with at least the alpha chain of FcεRI, but best if co-transfected with the human gamma chain, as this results in higher surface expression (Ali et al. 2019) (**B**). RBL cells can also be tailored to bind equine (Sabban et al. 2013) or canine (Ye et al. 2014) IgE, and probably many other mammalian species. Cells are incubated overnight with IgE-containing sera to be tested, which increases the surface density of the receptor (Yamaguchi 1997). The next day, the diluted serum is washed away, removing any unbound IgG (**C**) or other potential sources of interference. RBL cells are known to constitutively express two low-affinity IgG receptors, FcγRIIB (CD32b) and FcγRIII (CD16) (Boček et al. 1995). While the former has an intracellular tyrosine inhibitory motif (ITIM) and is thus incapable of activating the reporter cell line, the latter has an intracellular tyrosine activating motif (ITAM) but can only be activated by immune complexes due to its low affinity for IgG (Boček et al. 1995). Therefore, although it is currently unknown to which extent human IgG can bind to rat FcγRIIB and FcγRIII, most if not all of the IgG will be removed during the washes before the addition of the diagnostic allergen, avoiding any possible activation via IgG. Some sera can be cytotoxic to RBL cells, requiring a 1:100 dilution or a short thermal inactivation (5 min at 56°C), while other sera can be used, e.g., at 1:10 dilution without any pretreatment. The allergen is then added in a suitable concentration (usually in the range 0.1–1 μg/mL) and allowed to activate the sensitized reporter cells for various amounts of time, depending on the reporter gene used. Incubation times are shortest (45 min) for the NPY-mRFP RBL reporter (Barwary et al. 2020), which releases preformed fluorescent NPY-protein from the granules, 3–4 h for the RS-ATL8 (Nakamura et al. 2010), in which luciferase expression is induced, and 10–18 h (or longer if desired) for the NFAT-DsRed reporters (Wang et al. 2013), leading to the synthesis of red fluorescent protein in the cytosol in case of successful activation. In all cases, the high affinity of the receptor alpha chain for IgE (K_A_ ≥ 10^10^ M^−1^) and slow dissociation rate ensures that IgE in the serum sample is efficiently bound by the cells, while the natural cellular signal transduction machinery provides powerful multi-tiered signal amplification, in combination with sensitive reporters (luciferase or fluorescent protein), providing further amplification. This combination makes such IgE reporters highly efficient in detection of small amounts of allergen-specific IgE.

Hence, not only can parasite-specific IgE be formed as early as one month after (primary) infection, the absence of detectable IgE levels in serum in the presence of basophil sensitization points to another relatively simple, but currently underexploited fact: That a cellular readout for parasite-specific IgE is possibly superior to traditional methods (such as ELISA) in terms of sensitivity. As will be explained below, IgE crosslinking by matching allergens induces a powerful and fast multi-tiered cellular signal transduction cascade, in which a relatively modest engagement of a small percentage of IgE receptors on the surface results in a full cellular response within minutes after activation (Falcone et al. 2000) (Fig. 1). In the case of the IgE reporter systems, sensitivity is further enhanced, e.g., by the use of sensitive enzymatic reactions (e.g., luciferase, which adds another level of signal amplification).

When measuring IgE using ELISAs, competition of IgE with IgG, contained in much higher amounts in serum for the same epitopes, affects sensitivity, unless special measures for removal of IgG are taken (Kadooka et al. 2000). As will be discussed below, when using IgE reporter cell lines for IgE detection, such IgG removal is an inherent step of the protocol, as cells sensitized with IgE-containing serum overnight are washed before the activating antigen/allergen is added (Wan et al. 2014b). IgE is the isotype with the lowest concentration in serum. The reference range for total IgE concentrations in serum (e.g., 2–214 IU/mL (Martins et al. 2014)) depends on the population considered and the age of the individuals, but is, in any case, up to four or five orders of magnitude lower than total IgG levels. However, parasite-specific and total IgE levels can be considerably increased in parasitic infections, e.g., 1360 ± 721 U/mL in urinary schistosomiasis or 2355 ng/mL (~973 IU/mL) in filarial tropical eosinophilia (Neva et al. 1975).

## Use of specific IgE for detection of parasite infection

Despite the ubiquity of IgE responses in parasitic infection, not many authors have attempted to develop diagnostic technologies based on parasite-specific IgE detection. Notably, work from the laboratory of Shelley F. Walton has looked into IgE recognition of *Sarcoptes scabiei* antigens (Arlian et al. 2004; Walton et al. 2010), identifying a cysteine protease (Sar s 1), a serine protease (Sar s 3), and glutathione-S-transferase (Sar s 8) as allergens (Dougall et al. 2005). The authors suggested the use of apolipophorin Sar s 14.3 as a diagnostic allergen using dissociation-enhanced lanthanide fluorescent immunoassays (DELFIA), resulting in 100% sensitivity and 93.75% specificity. More importantly, the Der p 14 homolog apolipophorin from dust mites did not appear to be cross-reactive, which would have severely limited the usefulness of the diagnostic method. However, the most recent guidelines for the diagnosis of human scabies published in 2020 do not recommend or include any serological methods for diagnosis (Engelman et al. 2020). Apart from the description of tropomyosin and paramyosin as immunoreactive allergens (Naz et al. 2017), no serological tests are available for human scabies infection (Arlian et al. 2015). Human scabies serological diagnosis, thus, remains problematic (Walton and Currie 2007). The high sensitivity and specificity of the aforementioned IgE-based scabies test can, however, be used to illustrate the potential of using IgE for the diagnosis of parasite infection.

The diagnostic potential of parasite-specific IgE has also been explored in toxoplasmosis, as reviewed by Matowicka-Karna and Kemona (2014). Sensitivities reported in the reviewed studies ranged between 63% and 86.6%, with IgE levels detected very early and persisting for half a year or longer. We speculate here that used in combination with IgE reporter systems, sensitivity could be strongly enhanced. A recent IgE-based ELISA for diagnosis of *Strongyloides stercoralis* infection in humans based on a recombinant allergen (rA133) demonstrated very high specificity (99.3%) and sensitivity (100%), although tested serum numbers were relatively small (Ahmad et al. 2021).

## Humanized IgE reporter systems are extremely sensitive

While rat basophilic leukemia cells stably transfected with the human IgE receptor have been used for many years (Falcone et al. 2015), the last decade has seen the rather cumbersome and insensitive beta-hexosaminidase determination replaced by fluorescent or chemiluminescent reporter assays. These powerful IgE reporter cell lines can be used in a variety of formats, ranging from 384-well plates (Ali et al. 2017) to protein arrays (Kalli et al. 2020). The prototype, and to date still best performing IgE reporter system, was created by Ryosuke Nakamura and colleagues, who stably transfected a luciferase reporter into the humanized rat basophilic leukemia cell line RBL SX-38 (Nakamura et al. 2010), resulting in a cell line called RS-ATL8. RS-ATL8 cells are extremely sensitive, allowing detection of as little as 15 pg/mL of IgE, 0.04 to 0.4 ng/mL allergen-specific IgE, or as little as 1 fg/mL of egg allergen using sera from egg-allergic patients. We later were able to demonstrate the suitability of RS-ATL8 cells in the context of *Schistosoma mansoni* infection (Wan et al. 2014a) and vaccine candidate screening (de Melo et al. 2019). Due to their unrivaled sensitivity, using IgE reporter systems for detection of specific IgE has many advantages but also some drawbacks, both summarized in Table 1.

**Table 1:** Summary of advantages and disadvantages or IgE reporter cell lines for IgE-based serodiagnosis of parasitic infection

IgE-based serodiagnosis using reporter cell lines		
Advantages	Potential drawbacks	Possible solution or comments
RBLs are well studied, easy to grow, and are available in fluorescent (RFP) as well as chemiluminescent (luciferase) formats	RBLs do not bind human IgE	Stable transfection with human high-affinity IgE receptor
High sensitivity due to cellular signal transduction and high sensitivity of reporter signal (fluorescence, chemiluminescence)	High cost of luciferase substrate	Use of fluorescent IgE reporter systems (NFAT-DsRed, NPY-mRFP) which do not require any substrate. Creation of a novel cell line which produces the luciferase substrate autonomously *in situ* (i.e., intracellularly)
Fluorescence-based reporter (NFAT-DsRed) suitable for use in multiwell and array format	Luciferase-based RS-ATL8 not suitable for use in array format	Luciferase-based RS-ATL8 assay can be used in 96-well and 384-well plate format
Antigen binding by IgE not masked by IgG, IgM, or IgA competing for the same epitopes		Humanized IgE reporter RBL cell lines do not bind human IgG or IgM; this is removed during washing steps. RBL cells only express low-affinity IgG receptors (see Fig. 1C)
Inability of cross-reactive carbohydrate determinants (CCDs) to result in false-positive tests		Most cross-reactive carbohydrate determinants (CCDs) do not have the ability to crosslink FcεRI receptor-bound IgE; hence, IgE-recognizing CCDs is not detected in IgE reporter assays
	Insufficient knowledge of parasite allergens that could be used for diagnosis	IgE reporter systems can be used for the identification and characterization of parasite allergens
	Not amenable to lateral flow/rapid detection test format	Fluorescent reporter systems can be combined with allergen arrays, enabling high numerical power; however, this is not available for field testing and remains a lab-based diagnostic technology
	IgE is very low in serum compared with other Immunoglobulins	Reporter cell lines provide a natural signal amplification cascade; only a small percentage of IgE molecules need to be crosslinked to achieve full activation; use of a sensitive readout such as chemiluminescence (luciferase) or fluorescence RFP).
	High cross-reactivity of pan-allergens	Avoidance of pan-allergens, e.g., tropomyosin with known cross-reactivity with common environmental or food allergens (e.g., dust mites, crustaceans), as diagnostic allergens
	Recognition of allergens is genetically restricted	Use of a combination of diagnostic allergens
IgE in serum has a shorter half-life than IgG and disappears soon after infection		

A key confounding factor to consider is the high potential for cross-reactivity between parasitic and other (environmental or food) allergens. In the case of scabies, the occurrence of cross-reactive antibodies against house dust mites as confounding factor has been highlighted (Arlian et al. 2015). From this point of view, a diagnostic technology based on the detection of IgE should better avoid pan-allergens such as parvalbumins/polcalcins (Pritchard et al. 2020), tropomyosin or profilins (Hauser et al. 2010), and favor allergens that are unique to the parasite in question, or with restricted cross-reactivity, if at all possible. Bearing in mind the evolutionary hypothesis that the IgE response has evolved (at least in part) to protect the mammalian host against parasitic infection (Pritchard et al. 2020), which would favor recognition of ‘archetypal’ allergenic motifs, finding suitable allergens unique to a specific parasite may be a difficult proposition. However, it may still be possible to exploit subtle differences between the epitopes recognized by IgE.

*Anisakis simplex* needs to be discussed here briefly as a special case in parasitology and allergology, as this parasite plays a dual role as a zoonotic cause of infection (anisakiasis), and to a much larger extent also as a source of food allergens (Daschner et al. 2012). It is for its role in food allergy that *A. simplex* is the best-studied parasite in terms of its allergenicity, with at least 23 different allergens currently listed on the Allergome database (Mari et al. 2009). However, the high incidence of allergic sensitization due to ingestion of parasitized fish (Mazzucco et al. 2018), in addition to the known antigenic cross-reactivities with ascaridoid nematodes (Kennedy et al. 1988), also makes it highly unlikely that IgE-based detection can become a viable option for diagnosis of *A. simplex* infection.

Another potential issue, which is known to limit the clinical value of IgE-based diagnosis in allergy, is the existence of IgE directed against so-called cross-reactive carbohydrate determinants (CCDs) (Homann et al. 2017). CCDs are carbohydrate structures widely shared across allergens, which are the target of an IgE response. The most prominent example of CCD is galactose-α-(1,3)-galactose (α-Gal), which is the determinant underlying red meat allergy (Steinke et al. 2015). In this syndrome, individuals develop an IgE response specific for α-Gal, a carbohydrate linkage found in all mammals with the exception of higher apes and humans (Commins and Platts-Mills 2010), usually after sensitization due to repeated tick bites. This recently elucidated food allergy challenges current paradigms for several reasons (Iweala et al. 2020): (1) It is IgE-mediated, a reaction pattern usually classified as type I immediate hypersensitivity; however, symptoms in allergic patients occur several hours after red meat ingestion (Commins et al. 2016), making it the first example of a delayed-type, IgE-mediated hypersensitivity; (2) the IgE is directed against a carbohydrate rather than protein determinant, but unlike most CCDs, it is still able to elicit clinical symptoms; and (3) from the parasitological point of view, as sensitization is linked to exposure to certain ticks and, as shown by us and others recently, to *Ascaris lumbricoides* (Wilson et al. 2021) (Murangi et al. 2021), it may represent the first example of food allergy induced by direct exposure (in contrast to ingestion) to parasites. IgE sensitization to α-Gal induced disease may also go beyond food allergy, as suggested by a recent work implying a link with cardiovascular disease (Wilson and Platts-Mills 2019). The important fact to consider in the context of this article is that α-Gal sensitization can be detected using basophil activation assays, as recently shown by several authors (Commins et al. 2014; Hilger et al. 2016; Mehlich et al. 2019). However, we have been unable to detect activation of RS-ATL8 cells using sera of red meat allergic patients for reasons that still elude us.

Another important potential source of false-positive results in conventional serological methods caused by CCDs is the occurrence of a high level of cross-reactivity between parasite- and plant-derived glycans recognized by IgG, as shown for *Schistosoma mansoni* in several studies by Michael J. Doenhoff and his co-workers (El-Faham et al. 2020; Igetei et al. 2017, 2018). However, the relevance of these findings for IgE responses has yet to be examined.

With the exception of α-Gal-carrying allergens, the inability of most CCDs to crosslink receptor-bound IgE means that such allergens are unlikely to cause false-positive results in IgE reporter cell line assays, in contrast to assays measuring binding of specific IgE to allergens immobilized on a solid phase (Foetisch et al. 2003). Conversely, however, the observation that a large amount of IgE in Leishmaniasis appears to be directed against carbohydrate epitopes (Atta et al. 2004) implies that any leishmanial diagnostic allergens for use in IgE reporter systems, relying on effective IgE crosslinking by allergens, will have to be chosen carefully.

Taken together, parasite-specific IgE, despite its ubiquity in the immune response to parasites, is currently under-used as a diagnostic tool, in contrast to the use of allergen-specific IgE in allergic diseases, where it plays a central role. The development of highly sensitive IgE reporter systems may play an important role in overcoming some of the real or perceived issues limiting the diagnostic use of IgE in parasite infections. However, implementing an IgE-based diagnostic system will require a careful choice of suitable allergens. Only then can the performance of such diagnostic systems be tested in a variety of parasitic disease settings.

## Abbreviations

CCD: cross-reactive carbohydrate determinant
DELFIA: dissociation-enhanced lanthanide fluorescent immunoassays
ITAM: intracellular tyrosine activating motif
ITIM: intracellular tyrosine inhibitory motif
mRFP: monomeric red fluorescent protein
NFAT: nuclear factor of activated T-cells
NPY: neuropeptide Y
RBL: rat basophilic leukemia cell line
